# The efficacy of using 3D printing models in the treatment of fractures: a randomised clinical trial

**DOI:** 10.1186/s12891-019-2448-9

**Published:** 2019-02-08

**Authors:** Chunhui Chen, Leyi Cai, Wenhao Zheng, Jianshun Wang, Xiaoshan Guo, Hua Chen

**Affiliations:** Department of Orthopaedics, The Second Affiliated Hospital and Yuying Children’s Hospital of Wenzhou Medical University, 109 Xue Yuan Xi Road, Wenzhou, 325000 Zhejiang China

**Keywords:** 3D printing, Distal radius fracture, Feasibility, Orthopaedics, Surgery

## Abstract

**Background:**

The aim of this study was to evaluate the efficacy of the use of three-dimensional (3D) printing models for preoperative planning in cases of complex fracture.

**Methods:**

In total, 48 patients with AO type C fractures of the distal radius were enrolled in the study between January 2014 and January 2015. They were divided randomly into 3D model (*n* = 23) and routine treatment (*n* = 25) groups. A 3D digital model of each distal radius fracture in the former group was constructed. The model was exported to a 3D printer for construction of a full solid model. During each operation, the operative time, amount of blood loss, and frequency of intraoperative fluoroscopy were recorded, which were regarded as primary outcome measures. Patients were followed to evaluate surgical outcomes by Gartland–Werley scores, radiological evaluation, and range of motion of wrist, and these were regarded as the secondary outcome measures. In addition, we invited surgeons and patients to complete questionnaires.

**Results:**

The treatment of complex fractures using the 3D printing approach reduced the frequency of intraoperative fluoroscopy, blood loss volume, and operative time, but did not improve postoperative function compared with routine treatment. The patients wanted the doctor to use the 3D model to describe the condition and introduce the operative plan because it facilitated their understanding. The orthopaedic surgeons thought that the 3D model was useful for communication with patients, but were much less satisfied with its use in preoperative planning.

**Conclusion:**

Our study revealed that 3D printing models effectively help the doctors plan and perform the operation and provide more effective communication between doctors and patients, but can not improve postoperative function compared with routine treatment.

**Trial Registration:**

This trial was registered at the Chinese Clinical Trial Registry on May 9, 2017 (ChiCTR-IRP-17011343, http://www.chictr.org.cn/showproj.aspx?proj=19264).

## Background

Many studies have described the use of three-dimensional (3D) printing models in the treatment of orthopaedic diseases [1–4]. A PubMed search conducted on April 8, 2017, using the keywords ‘3D printed’, ‘fracture’, and ‘orthopaedics’ yielded a total of 684 publications indexed between 2013 and 2016 (35 articles in 2013, 108 articles in 2014, 226 articles in 2015, and 315 articles in 2016). These results reveal a nine-fold increase in publication on this topic between 2013 and 2016, but the efficacy of use of this method in clinical practice remains unclear. The literature also reveals a lack of randomised controlled trials evaluating the efficacy of this technological application. In this study, we evaluated the use of 3D printing medols in the treatment of fractures, taking distal radius fracture as an example.

Distal radius fracture is one of the most common upper-extremity injuries [5], which represent about 17% of all skeletal fractures [6, 7]. Most distal radius fractures result in > 2 mm fragment displacement, commonly accompanied by the presence of multiple fragments on articular surfaces, which represents a great challenge for orthopaedic surgeons [8]. The efficacy of routine surgical procedures, such as closed reduction with mini-external fixation, percutaneous fixation with K-wires, and open reduction and internal fixation with a volar locking or non-locking plate, is insufficient due to complications such as malunion, subluxation, and late fracture collapse [9–12]. Due to the high incidence of distal radius fracture and the complications associated with standard treatments, a more scientific approach to the treatment of these fractures is needed. Some authors have reported that the use 3D printing models in the treatment of fractures has beneficial effects [13, 14]. Thus, we assumed that 3D printing models could be applied as a novel approach in the treatment of distal radius fractures. As the anatomical volumes of distal radius are small, a realised model can be created conveniently, with little time and cost expenditure. In addition, the high incidence of such fractures enables the collection of data on standard cases. Thus, we believe that distal radius fracture is a suitable example with which to evaluate the efficacy of use of 3D printing models in the treatment of fractures.

Our aim was to use 3D printing models to reconstruct the distal radius fractures in patients and evaluate its efficacy in the surgical outcomes for the fracture repair and in the communication between doctors and patients. We assumed that 3D printing models effectively help the doctors plan the operation and surgical outcomes, and provide more effective communication between doctors and patients.

## Methods

### Study design

This randomised, single-blinded, prospective clinical trial was conducted to evaluate the efficacy of using 3D printing in the treatment of fractures. The Research Ethics Boards of our university approved this study. This randomised clinical trial was registered with the Chinese Clinical Trial Registry. Written informed consent was obtained from all participants following a detailed description of the study’s purpose; all participants agreed to the publication of relevant demographic and clinical features of their cases. This study was performed in accordance with national and local laws and guidelines. Our report does not contain information or images that could lead to the identification of study participants.

#### Study population

All patients with AO type C fractures were enrolled in the study between January 2014 and January 2015. Information of patients, such as sex, age, Cause of injury, and AO classification, was recorded. Inclusion criteria were adult patients (aged 18 years and above) with a closed AO type C distal radius fracture Patients. Patients with complications, such as nerve and/or vascular injuries, infection, polytrauma, or open fracture, were excluded.

### Study randomization

Participants were divided randomly into 3D model and routine treatment groups. The fourth author of this report who was not involved in clinical treatment divided randomly participants by coin toss. Patient age and sex, time from injury to surgery, and cause of injury did not differ significantly between groups (Table 1). All patients agreed to a minimum follow-up period of 1 year after surgery.

**Table 1:** Patients’ general conditions

		3D model group	Routine treatment group
*N*		23	25
Sex	Male	14	17
	Female	9	8
Age (years)		38.7 ± 13.6	40.7 ± 11.4
Cause of injury	Traffic accident Heavy blow Fall from height	18 4 1	17 6 2
AO classification	C1 C2 C3	10 9 4	11 11 3
Time from injury to operation (days)		3.3 ± 1.8	3.7 ± 1.6
Follow-up duration (months)		13.0 ± 0.7	13.1 ± 0.7

### Study interventions

**Fig. 1:**
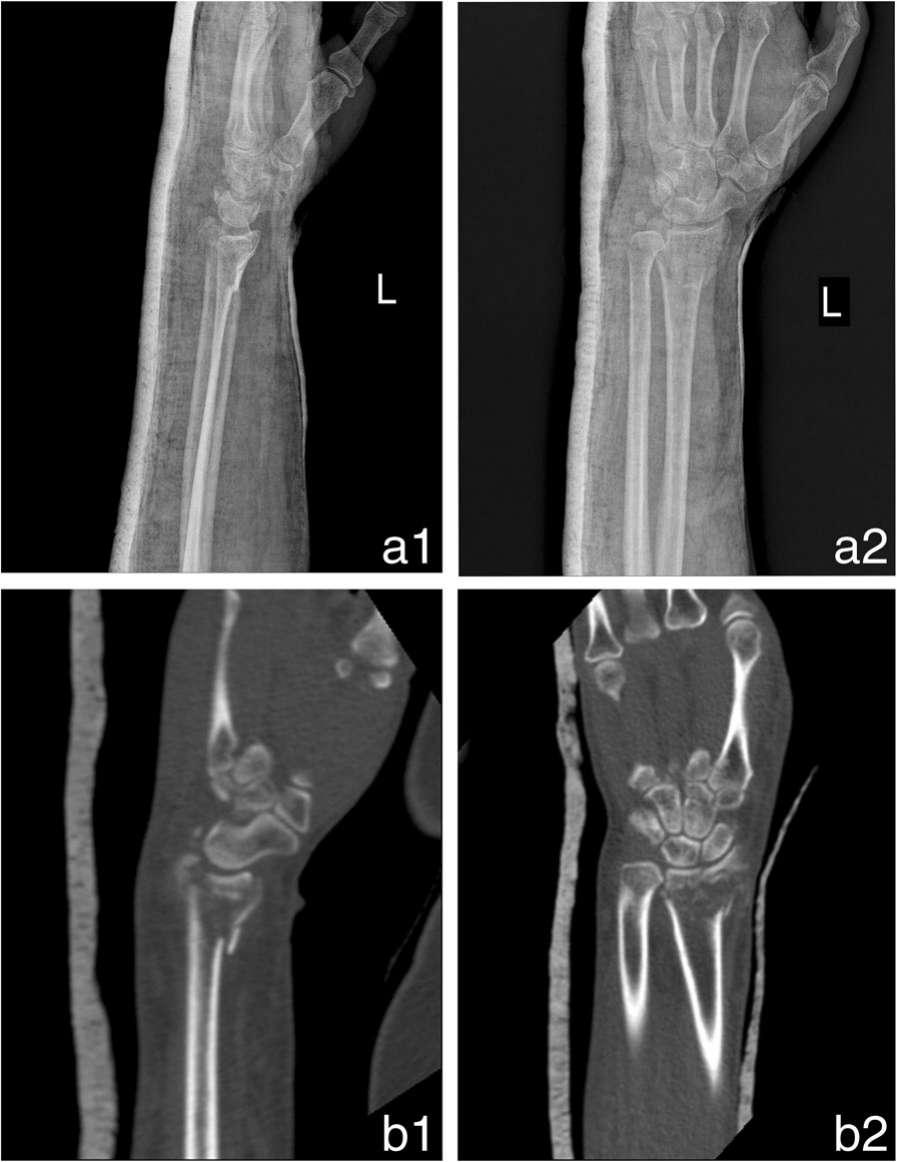
A 41-year-old man patient had a distal radius fracture, classified as AO type C1, selected as a typical case. Preoperative radiological characteristics of a distal radius fracture. **a1, a2:** Anteroposterior and lateral X-ray. **b1, b2:** Computed tomography (CT) images of the fracture

**Fig. 2:**
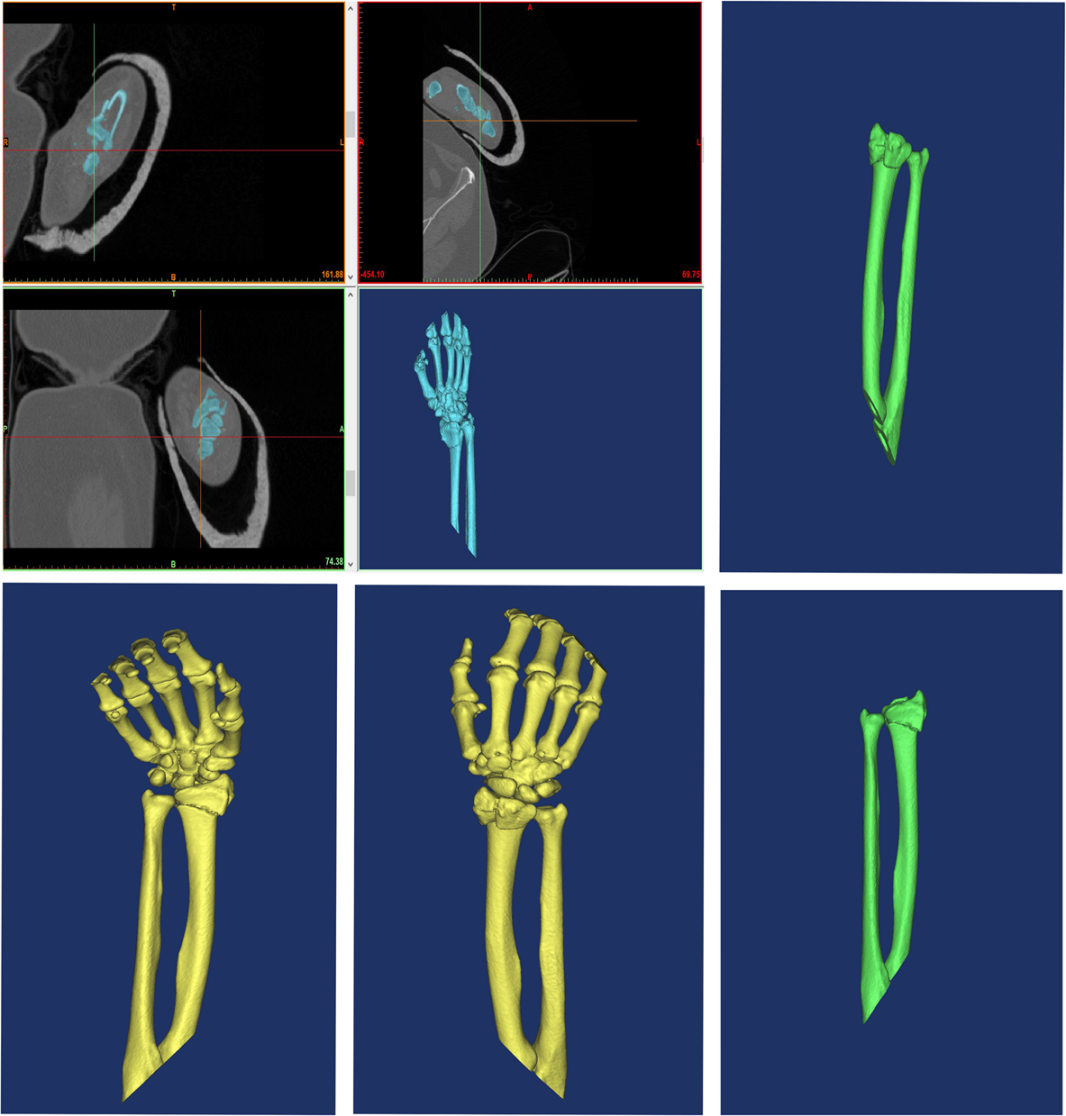
Reconstruction of a distal radius fracture in Mimics software v10.01

**Fig. 3:**
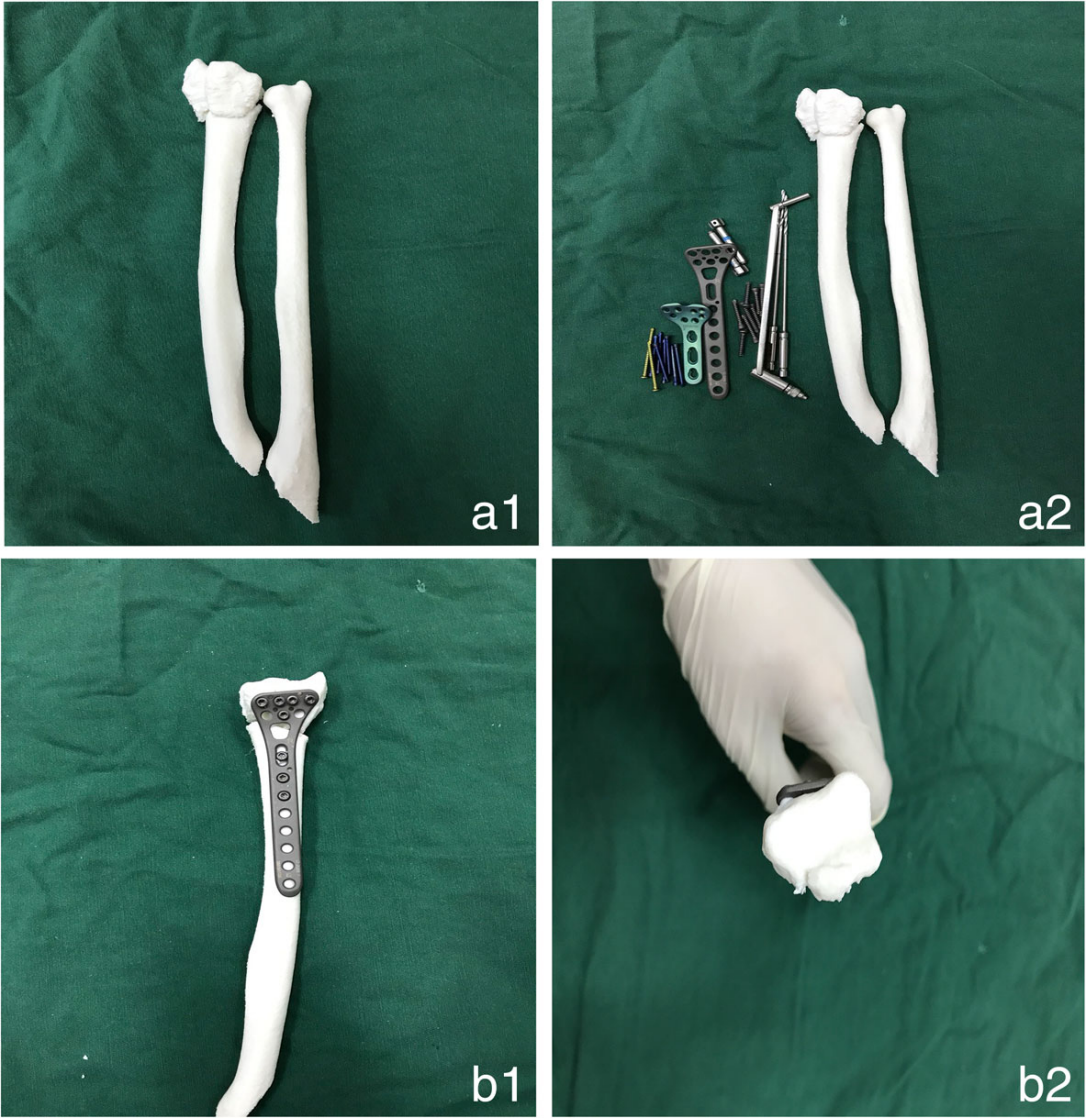
Preparation and outcomes of the surgical simulation. a**1:** The solid 3D model. **a2:** Preparation of the surgical simulation. **b1, b2:** Outcomes of the surgical simulation

We received computed tomography (CT) scans of distal radius fractures, obtained in our hospital using the Star PACS system (INFINITT, Seoul, South Korea). The original CT data were stored in DICOM format; 3D images were reconstructed using Mimics software (version 10.01; Materialise, Leuven, Belgium). The threshold value was set at ‘Bone (CT) 226-Max’, which is optimal for bone reconstruction. We exported the reconstructed data to the 3D printing software in STL format. 3D digital models were created, saved in Gcode format, and exported to a 3D printer (3D ORTHO; Waston Med Inc., Changzhou, Jiangsu, China) for construction of full solid models. Polylactic acid (3D ORTHO; Waston Med Inc.) was used as the printing material; several parameters were used to section the prototype. Processing parameters were: (1) 1.65-mm plastic filament diameter, (2) 0.3-mm layer height, (3) plastic extrusion at 210 °C, and (4) a 60-mm s^− 1^ plastic feed rate. In total, the manufacture of each fracture prototype took approximately 5 h (1 h pre-processing, 3–4 h printing). The 3D-printed models clearly showed the structural characteristics of the fractures, which aided in the design of surgical plans. On the 3D model, we split the fracture fragments according to the fracture line, and then temporarily reset the fragments with K-wire, followed by fixation of the fragments with metal plates and screws. In this way, the type and dimensions of the implant required were determined preoperatively, and we could choose suitable metal plates and screws. We also recorded the time required to reset each fracture. Each simulated surgery took approximately 2.5 h. Three surgeons performed internal fixation of the distal radius using the Henry approach [15] in patients in both groups. Figure 1, 2 and 3 showed the images of a typical case.

### Assessment of outcome

The main parameters including the duration of surgery, volume of blood loss, and frequency of intraoperative fluoroscopy were regarded as the primary outcome measures. The fifth author, who was not involved in clinical treatment, collected surgical outcome data based on the last follow-up. We assessed the final surgical outcomes using the Gartland–Werley scores, as modified by Chun and Palmer [16], radiological evaluation, and range of motion of wrist. Gartland–Werley scores, radiological evaluation, and range of motion of wrist were regarded as the secondary outcome measures.

### Questionnaire

We created simple questionnaires to examine surgeons’ and patients’ perspectives regarding the effectiveness of the 3D-printed prototype. All patients completed the patient questionnaire. We invited 30 surgeons who had used 3D printing models in the treatment of fractures to complete the questionnaire designed for medical professionals. Three of the 30 orthopaedic surgeons involved in this study, and other 27 orthopaedic surgeons were completely independent from the current study. Scores ranged from 1 to 10 points, with 1 indicating the worst evaluation and 10 indicating the best evaluation.

### Statistical analyses

Statistical analyses were performed using the IBM SPSS software (version 21.0; IBM Corp., Armonk, NY, USA). Qualitative variables were summarised as numbers and percentages, and quantitative variables were summarised as means with standard errors and ranges. We used the chi-squared test (or Fisher’s exact test when appropriate) for qualitative variables, and the *t* test (or Mann–Whitney test when appropriate) for quantitative variables. Statistical significance was set at *P* <  0.05.

## Results

### Study population

**Fig. 4:**
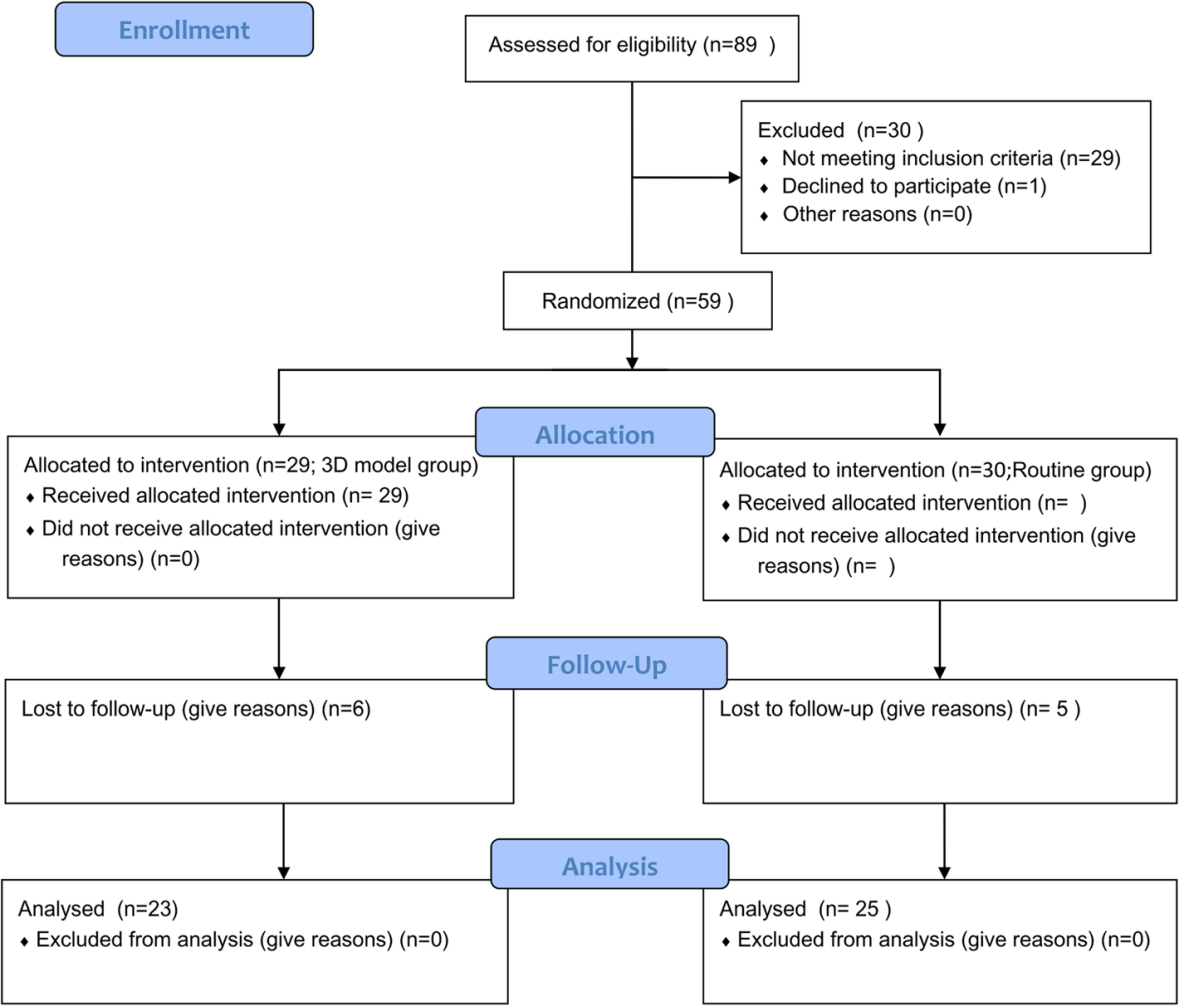
CONSORT 2010 Flow Diagram

AS the Fig. 4 (CONSORT 2010 flow diagram) shows, 89 patients were screened and identified as eligible;1 eligible patients declined to participate in either treatment group. Among the remaining 88 patients, 59consented to undergo randomization and 29 declined to undergo randomization. 29 patient who was randomly assigned to the 3D model group, 6 patient Lost to follow-up. 30 patient who was randomly assigned to the routine group, 5 patient Lost to follow-up. Finally, 23 patients in the 3D model group and 25 patients in the routine group were enrolled.

### Primary outcome measures

All patients were followed for at least 12 months, and data from the last follow-up were used to determine final outcomes. The duration of follow-up did not differ between the routine treatment and 3D model groups (13.1 ± 0.7 vs. 13.0 ± 0.7 months, Table 1). The operative time, amount of blood loss, and frequency of intraoperative fluoroscopy were regarded as primary outcome measures. The results were shown in Table 2. The mean operative time was significantly shorter in the 3D model group than in the routine treatment group (66.5 ± 5.3 vs. 75.4 ± 6.0 min, *P* <  0.001). Blood loss volume differed significantly between the 3D model and routine treatment groups (41.1 ± 7.5 vs. 54.2 ± 7.9 mL, *P* <  0.05). The mean frequency of fluoroscopy was significantly greater in the routine treatment group than in the 3D model group (5.6 ± 1.6 vs. 4.4 ± 1.4 times, *P* = 0.011).

**Table 2:** Results of primary outcome measures

Group	*n*	Operation time (min)	Blood loss (mL)	Frequency of intraoperative fluoroscopy (*n*)
Routine treatment	25	75.4 ± 6.0	54.2 ± 7.9	5.6 ± 1.6
3D model	23	66.5 ± 5.3	41.1 ± 7.5	4.4 ± 1.4
*T*	–	5.427	5.889	2.644
*P*	–	< 0.001	< 0.001	0.011

### *Secondary outcome* measures

Gartland–Werley scores, radiological evaluation, and range of motion of wrist were regarded as the secondary outcome measures. The results were shown in Table 3 and Table 4. The mean Gartland–Werley scores did not differ significantly between the 3D model and routine treatment groups (75.7 ± 15.5 vs. 74.8 ± 16.6, *P* = 0.211; Table 3).

**Table 3:** Results from radiological condition and Gartland–Werley scores

Group	Gartland–Werley scores	Ulnar Deviation (Degree)	Palmar tilt (Degree)	High of radial styloid process(mm)
Routine group	74.8 ± 16.6	20.4 ± 1.5	12.7 ± 1.9	12.6 ± 1. 8
3D model group	75.7 ± 15.5	20.9 ± 1.7	12.2 ± 1.5	12.6 ± 1.9
*T*	0.211	1.030	0.927	0.016
*P*-value	0.211	0.309	0.359	0.987

**Table 4:** Differences in postoperative range of motion of wrist compared with the healthy wrist

Group	Extension(°)	Flexion (°)	Pronation (°)	Supination (°)
Routine treatment	3.8 ± 3 .1	3.6 ± 2.7	4.5 ± 3.7	4.9 ± 3.3
3D model	4.1 ± 3.5	3.1 ± 2.7	5.1 ± 3.2	4.4 ± 3.3
*T*	0.301	0.663	0.605	0.509
*P*	0.765	0.511	0.548	0.613

Postoperative radiological evaluation was performed in both groups. The degree of ulnar deviation was 20.4 ± 1.5° in the routine treatment group and 20.9 ± 1.7° in the 3D model group. The degree of palmar tilt was 12.7 ± 1.9° in the routine treatment group and 12.2 ± 1.5° in the 3D model group. The height of the radial styloid process was 12.6 ± 1.8 mm in the routine treatment group and 12.4 ± 1.9 mm in the 3D model group. No significant difference in ulnar deviation, palmar tilt, or radial height was observed between groups (*P* > 0.05; Table 3).

Postoperative range of motion of wrist was observed in both groups (Table 4). Compared with the healthy wrist, the average difference in extension was 3.8 ± 3.1° in the routine treatment group and 4.1 ± 3.5° in the 3D model group. The average difference in flexion was 3.6 ± 2.7° in the routine treatment group and 3.1 ± 2.7° in the 3D model group. The average difference in pronation was 4.5 ± 3.7° in the routine treatment group and 5.1 ± 3.2° in the 3D model group. The average difference in supination was 4.9 ± 3.3° in the routine treatment group and 4.4 ± 3.3° in the 3D model group. No significant difference in extension, flexion, pronation, or supination was observed between groups (*P* > 0.05; Table 4).

### Questionnaire findings

Scores for the questionnaire items ‘how much do you know about your fracture situation’ and ‘how much do you know about your surgical plan’ differed significantly between patients in the routine treatment and 3D model groups (*P* = 0.001; Table 5). Among surgeons, the mean score for ‘usefulness of the 3D prototype for communication with patients’ was 9.1 ± 0.8 and that for ‘overall usefulness of 3D printing models’ was 6.7 ± 1.4.

**Table 5:** Questionnaires for patients and doctors

Question	Subjective field (for patients)	Average score	*P*
1	How much do you know about your fracture situation? (Routine treatment group)	5.1 ± 2.1	0.000
2	How much do you know about your fracture situation? (3D model group)	7.6 ± 1.6	
3	How much do you know about your surgical plan? (Routine treatment group)	5.4 ± 1.9	0.001
4	How much do you know about your surgical plan? (3D model group)	7.3 ± 1.8	
5	How much did the 3D prototype help you to obtain a clear understanding of your condition?	91 ± 0.5	–
6	How much would you like the doctor to use a 3D prototype to communicate with you about your condition?	9.3 ± 0.5	–
	Subjective field (for doctors)		
1	Degree of verisimilitude of the 3D prototype to the actual fracture	8.7 ± 1.4	
2	Usefulness of the 3D prototype for preoperative planning	5.9 ± 1.6	
3	Usefulness of the 3D prototype for communicating with patients	9.1 ± 0.8	
4	Overall usefulness of 3D printing models	6.7 ± 1.4	
5	Would you use 3D printing models to treat a complex fracture again?	Yes = 8 No = 22	

## Discussion

3D printing technology is developing rapidly in the field of orthopaedic surgery, and some scholars have published on its applications [17–22]. They maintain that 3D printing models can make diagnosis and surgery more directly visible, realistic, and specific by assisting in the clinical diagnosis, aiding the planning of complex operation strategies, and allowing simulation of the operation, rendering the use of this method in orthopaedic surgery feasible and accessible. Because 3D printing can be used to produce an individualised, realised solid prototype of a fracture before complex surgery, junior surgeons can observe the anatomical structure of the fracture and simulate the surgical operation to determine the size of the implant required for internal fixation.

However, whether this technological application will become an effective method in clinical practice remains unknown. The results of our study suggest that the use of 3D printing models reduced the operation time, volume of blood loss, and frequency of intraoperative fluoroscopy, but did not improve postoperative function. In our study, the operative time was 9 min shorter (*P* <  0.05) and the volume of blood loss was 10 mL less (*P* < 0.05) in the 3D model group than in the routine treatment group. It confirmed that the fracture prototype can increase the surgery efficiency and reduce the operation time. Our study also revealed a few limitations of this technology; it models only the bones, without considering the impact of soft tissue, which differs greatly from the real operative situation. In addition, 3D printing involves processing by software and machine production, which introduces errors that may affect the authenticity of the model.

The questionnaire results revealed that the patients wanted the doctor to use the 3D model to describe their condition and introduce the operative plan, and that the condition and operative plan were more likely to be understood in the 3D model group than in the routine treatment group (*P* < 0.05). Overall satisfaction with and perceived usefulness of the 3D prototype were greater among patients than among surgeons. The orthopaedic surgeons felt that the 3D model was useful for communication with their patients, but were much less satisfied with its use for preoperative planning. Only eight doctors reported that they would be willing to use 3D printing models to treat complex fractures again; most surgeons were thus reluctant to use the 3D printing technique in the treatment of fractures. The primary reason for this reluctance is probably the time required for simulated surgery. A second reason may be that the bone model does not consider the impact of soft tissue, which differs from the real operative situation. A third reason may be that the imaging modality can only provide data from an accumulation of multiple CT slices, although these slices are 3 mm; error often occurs between slices. In addition, the 3D material is very different from real bone.

The use of 3D printing in the treatment of fractures also has several advantages. The fracture can be viewed on the prototype in 360°, enabling accurate characterisation and enhancing the doctor’s realistic and direct understanding of the patient’s condition. The model also helped patients and family members to understand the patients’ conditions, enabling more effective communication. Patients and family members were satisfied with the results of this communication method, which increased patient compliance during treatment. In addition, the frequent performance of operations, as well as practice and surgical simulation, are known to improve surgeons’ skills.

We would like to acknowledge several limitations of our study. Patient randomisation by coin toss is no longer considered to be a modern procedure because the coin or the flipper may be biased, which does not ensure 1:1 randomisation.

## Conclusions

Our study revealed that 3D printing models effectively help the doctors plan and perform the operation and provide more effective communication between doctors and patients, but can not improve postoperative function compared with routine treatment.

## Abbreviations

3D: Three-dimensional
CONSORT: Consolidated standards of reporting trials
CT: computed tomography
K-wire: Kirschner wire
PACS: Picture archiving and communication systems
STL: stereolithography
DICOM: Digital imaging and communications in medicine
